# Clinical Utility of Optical Genome Mapping and 523-Gene Next Generation Sequencing Panel for Comprehensive Evaluation of Myeloid Cancers

**DOI:** 10.3390/cancers15123214

**Published:** 2023-06-16

**Authors:** Nikhil Shri Sahajpal, Ashis K. Mondal, Harmanpreet Singh, Ashutosh Vashisht, Sudha Ananth, Daniel Saul, Alex R. Hastie, Benjamin Hilton, Barbara R. DuPont, Natasha M. Savage, Vamsi Kota, Alka Chaubey, Jorge E. Cortes, Ravindra Kolhe

**Affiliations:** 1 Greenwood Genetic Center, Greenwood, SC 29646, USA; nsahajpal@ggc.org (N.S.S.); bhilton@ggc.org (B.H.); dupont@ggc.org (B.R.D.); 2 Department of Pathology, Medical College of Georgia, Augusta University, Augusta, GA 30912, USA; amondal@augusta.edu (A.K.M.); hsingh1@augusta.edu (H.S.); avashisht@augusta.edu (A.V.); sananth@augusta.edu (S.A.); nsavage@augusta.edu (N.M.S.); vkota@augusta.edu (V.K.); 3 Bionano Genomics Inc., San Diego, CA 92121, USA; dsaul@bionanogenomics.com (D.S.); ahastie@bionanogenomics.com (A.R.H.); achaubey@bionanogenomics.com (A.C.); 4 Department of Medicine, Georgia Cancer Center, Medical College of Georgia, Augusta University, Augusta, GA 30912, USA; jorge.cortes@augusta.edu

**Keywords:** optical genome mapping, 523-gene NGS panel, myeloid cancers

## Abstract

**Simple Summary:**

The current standard-of-care (SOC) genetic testing of myeloid cancers in limited by resolution and/or is targeted to investigate limited regions/genes in the genome. As a result, these genomes remain poorly characterized and warrant further investigation using emerging technologies. In this study, we have utilized a novel approach of combining a high-resolution cytogenetic technology with next-generation sequencing to obtain a comprehensive genomic profile of these tumors. This approach identified genetic alterations in previously negative cases and identified clinically relevant alteration in additional cases. This combinatorial approach has the potential to replace the current SOC workflow.

**Abstract:**

The standard-of-care (SOC) for genomic testing of myeloid cancers primarily relies on karyotyping/fluorescent in situ hybridization (FISH) (cytogenetic analysis) and targeted gene panels (usually ≤54 genes) that harbor hotspot pathogenic variants (molecular genetic analysis). Despite this combinatorial approach, ~50% of myeloid cancer genomes remain cytogenetically normal, and the limited sequencing variant profiles obtained from targeted panels are unable to resolve the molecular etiology of many myeloid tumors. In this study, we evaluated the performance and clinical utility of combinatorial use of optical genome mapping (OGM) and a 523-gene next-generation sequencing (NGS) panel for comprehensive genomic profiling of 30 myeloid tumors and compared it to SOC cytogenetic methods (karyotyping and FISH) and a 54-gene NGS panel. OGM and the 523-gene NGS panel had an analytical concordance of 100% with karyotyping, FISH, and the 54-gene panel, respectively. Importantly, the IPSS-R cytogenetic risk group changed from very good/good to very poor in 22% of MDS (2/9) cases based on comprehensive profiling (karyotyping, FISH, and 54-gene panel vs. OGM and 523-gene panel), while additionally identifying six compound heterozygous events of potential clinical relevance in six cases (6/30, 20%). This cost-effective approach of using OGM and a 523-gene NGS panel for comprehensive genomic profiling of myeloid cancers demonstrated increased yield of actionable targets that can potentially result in improved clinical outcomes.

## 1. Introduction

Myeloid malignancies are characterized by uncontrolled proliferation and/or defects in the differentiation of abnormal myeloid progenitor cells. Myelodysplastic syndromes (MDS) and myeloproliferative neoplasms (MPN) often progress to higher-grade myeloid malignancies, i.e., acute myeloid leukemia (AML) [1]. The National Comprehensive Cancer Network (NCCN) guidelines for the genetic diagnosis of MDS/MPN/AML recommends bone marrow cytogenetic analysis using karyotyping and fluorescent in situ hybridization (FISH), in addition to molecular analysis for at least *JAK2*, *CALR,* and *MPL* genes for MPN and *c-KIT*, *FLT3* (ITD and TKD), *NPM1*, *CEBPA* (biallelic), *IDH1*, and *IDH2* for AML. The guidelines further recommend molecular testing using multiplex gene panels and next-generation sequencing (NGS) analysis for comprehensive prognostic assessment [2].

In this context, the routine assessment of myeloid cancers in current clinical care incorporates karyotyping and FISH—occasionally chromosomal microarrays (CMA)—for cytogenetic analysis [2,3,4], and targeted gene panels (most frequently including ≤54 genes) that screen prominent hotspot pathogenic variants for molecular characterization [5,6]. This approach has been widely implemented but suffers from several limitations that include, first, the need of a combination of traditional cytogenetic methods (karyotype and FISH/CMA) to obtain structural variation analysis according to the guidelines, as each technology has limitations. Karyotyping is sufficient for genome-wide structural variation (SV) detection but has a lower limit of detection for abnormalities between 5–10 Mb. FISH can detect and clarify rearrangements below the resolution of karyotype but is not appropriate/feasible or genome-wide analysis. CMA has the best genome-wide resolution to detect copy number variations (CNV) but cannot detect balanced SV or determine the location or orientation of copy number gain (or amplification) regions of the genome. Second, the use of small-targeted gene panels for molecular characterization yields an incomplete sequence variant profile (omitting several of the growing number of important tumorigenic sequence variants) [7]. Despite this standard of care workflow including both cytogenetic and molecular methods, approximately 50% of myeloid cancers remain cytogenetically normal [8,9,10] and insufficient sequencing variant profiles are unable to resolve the heterogeneity in diagnostic features and/or outcomes in patients with myeloid cancers [5,6,7,11]. On multiple occasions, with our existing testing methods (karyotyping/FISH/CMA/54-NGS) patients with myeloid malignancies harboring increased blast percentage are found to have a normal genome profile in standard reports. This has consistently led to suboptimal diagnostic or prognostic classification and inadequate therapy/transplant selection, which ultimately leads to poor clinical outcomes. 

Recently, in the cytogenetic domain, optical genome mapping (OGM) has emerged as a next-generation cytogenomic technology that can detect several classes of SVs (insertions, deletions, duplications, inversions, translocations), and complex rearrangements at a higher resolution than the SOC methods [12,13,14,15,16,17]. The OGM technique is based on imaging ultra-long DNA (>150 kbp) molecules labelled at specific sequence motifs (CTTAAG) that span the entire genome (on average, every 5 kbp). Generation of OGM data at approximately 400x coverage allows for the detection of low-level SVs as required for many myelogenous malignancy samples since cancer cells will be analyzed through a background of stromal cells in blood and bone marrow. Recently, we, along with other groups, evaluated OGM potential and role in the detection of SVs in patients with hematological malignancies and found ~100% clinical concordance with traditional cytogenetic analysis [15,16,17,18]. In addition, OGM was able to provide higher resolution and resolve or refine previously identified aberrations as well as identifying additional clinically relevant abnormalities that remained beyond the purview of current methods [15,16,17,18]. 

In the molecular domain, targeted NGS panels (≤54 genes) are primarily employed as they generate high coverage (~500x) and are cost and time-effective with minimal data analysis and reporting complexity. Recently, whole-genome sequencing (WGS) at ~50–120x read depth analysis has been reported to identify targeted SVs, karyotype level CNVs, and sequencing variants in myeloid neoplasms [19]. Though it represents a significant advancement, currently WGS is time and cost-prohibitive requiring sophisticated instrumentation and bioinformatic (data analysis, interpretation, and reporting) challenges, and can report false-negative sequence variants compared to the 40-gene NGS panel [19]. For widespread utilization, a sequencing strategy should provide a thorough molecular characterization with minimal data analysis and enable easy reporting. In this regard, we have previously validated a 523-gene NGS panel for simultaneous analysis of sequence variants, tumor mutation burden (TMB), and microsatellite instability (MSI) for myeloid cancers [7]. 

We hypothesize that a combination of OGM and a 523-gene NGS panel would provide comprehensive characterization of cytogenetic and molecular variants in myeloid malignancies. In this study, we evaluated the performance and clinical utility of OGM and the 523-gene NGS panel for the comprehensive genomic profiling of 30 myeloid cancer samples and compared it to our current diagnostic workflow that includes SOC cytogenetic methods (karyotyping and FISH) and a 54-gene NGS panel. Finally, we demonstrate the visualization of OGM and NGS within the same bioinformatic platform to streamline analysis and reporting.

## 2. Materials and Methods

### 2.1. Sample Selection

**Figure 1:**
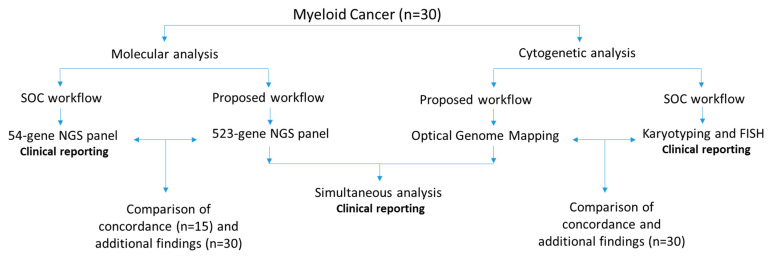
The study design shows the comparison of standard-of-care methods with proposed work using optical genome mapping and a 523-gene NGS panel.

In this retrospective study, 30 well-characterized bone marrow aspirate (BMA) samples received in our clinical laboratory for routine cytogenetic analysis (karyotype and FISH) and molecular analysis—54-gene myeloid panel (first 15 samples) or 523-gene NGS panel—were analyzed. All had abnormal cytogenetic and/or molecular profiles. Of these 30 samples, 9 were myelodysplastic syndrome (MDS), 16 were acute myeloid leukemia (AML), 4 were myeloproliferative neoplasms (MPN), and 1 was chronic myeloid leukemia (CML). These 30 samples were processed for OGM and the 523-gene NGS panel, with the technologist blinded to previous SOC results. The data were analyzed in a blinded fashion by the analyst (NSS), which were then compared for concordance with SOC methods by a board-certified laboratory director (RK). The SVs that were concordant were grouped under “concordant calls” and all other reported SVs were considered as additional findings. The study was approved by the IRB A-BIOMEDICAL I (IRB REGISTRATION #00000150), Augusta University (HAC IRB # 611298). Waiver of informed consent was granted by the IRB; all PHI was removed, and all data were anonymized before processing the samples (Figure 1).

### 2.2. Optical Genome Mapping

Ultra-high molecular weight (UHMW) DNA was isolated, labeled, and processed for analysis on the Bionano Genomics Saphyr^®^ platform following the manufacturer’s protocols (Bionano Genomics, San Diego, CA, USA). Briefly, a frozen BMA aliquot (650 μL) was thawed, and cells were counted using HemoCue (HemoCue Holding AB, Ängelholm, Sweden). Subsequently, a BMA aliquot comprising approximately 1.5 million nucleated white blood cells was centrifuged, the cells were digested with Proteinase K, and lysed using LBB buffer. DNA was precipitated on a nanobind magnetic disk using isopropanol and washed using buffers (buffers A and B). The UHMW-bound DNA was suspended in elution buffer and quantified using Qubit broad range (BR) dsDNA assay kits (ThermoFisher Scientific, San Francisco, CA, USA).

DNA labeling was performed following the manufacturer’s protocols (Bionano Genomics, San Diego, CA, USA) in which 750 ng of purified UHMW DNA was labeled at a specific 6-base sequence motif with DL-green fluorophores using direct labeling enzyme 1 (DLE-1) reactions. Following the labeling reaction, the DLE enzyme was digested using PK and the DL-green was removed in two steps using an adsorption membrane in a micro-titer plate. Finally, the DNA backbone was stained blue using DNA stain and quantified using Qubit high sensitivity (HS) dsDNA assay kits. Labeled DNA was loaded onto flow cells of Saphyr chips for optical imaging. The fluorescently labeled DNA molecules were imaged on the Saphyr platform after the labeled DNA molecules were electrophoretically linearized in the nanochannel arrays. Analytical QC targets were set to achieve >400X effective coverage of the genome, >70% mapping rate, 13–17 label density (labels per 100 kbp), and >230 kbp N50 (of molecules > 150 kbp). 

### 2.3. OGM Variant Calling and Data Analysis

Genome analyses were performed using Bionano Access (v.1.6)/Bionano Solve (v.3.6) software (Bionano Genomics, San Diego, CA, USA), and the rare variant analysis pipeline for all of the samples to assess and interrogate SVs and CNVs. Briefly, molecules of a given sample dataset were directly aligned to GRCH38, reference human genome assembly. SVs were identified where the pattern of labels in the molecules differed from the GRCH38 reference genome. Insertion, duplications, deletions, inversions, and translocations were called based on this alignment. SVs generated by the rare variant pipeline were then annotated with known canonical gene sets extracted from the reference genome assembly and compared to a control dataset (179 DLE-1-labelled samples available in Bionano access software) to estimate the population frequency of SVs. Additionally, a coverage-based algorithm was used to call large CNVs (>500 kbp). A standard operating procedure (SOP) was devised to enable analysts to systematically and efficiently select rare (<1% population frequency) variants with additional criteria that included filtering for variants overlapping genes. 

### 2.4. Analytical Comparison between OGM and SOC Results

Of the 30 myeloid neoplasms that had received cytogenetic (karyotyping and FISH), 8 were classified as complex cases (≥3 cytogenetic aberrations) and 22 were simple cases (<3 cytogenetic aberrations). Overall, within the 30 cases, a total of 72 aberrations had been reported. They included 31 aneuploidies, 16 deletions, 10 translocations, 5 insertions/duplication/additional material, 9 marker chromosomes, and 1 ring chromosome, which were used for comparison with the OGM results.

### 2.5. 523-Gene NGS Panel

DNA was isolated from BMA using the QIAamp DNA Blood Mini kit (QIAGEN, Hilden, Germany) as per the manufacturer’s protocol. Double-stranded DNA was measured using Qubit dsDNA broad-range assay kit (#Q32850, Invitrogen, Waltham, MA, USA) and 120 ng gDNA was used for library preparation. The libraries were prepared using the hybrid capture-based TSO 500 library preparation kit (#20028214, TruSight Oncology 500 DNA Kit, Illumina, San Diego, CA, USA) following the manufacturer’s instructions. In brief, the DNA was fragmented using an ultrasonicator (Covaris, Woburn, MA, USA) with a target peak of ~130 bp. After end repair, A-tailing, and adapter ligation, the adapter-ligated fragments were amplified using index PCR (UP-index) specific primers. Further, the libraries were enriched through a hybrid capture-based method using specific probes. This was followed by PCR-based enrichment, cleanup, and quantification of double-stranded DNA using high sensitivity Qubit (#Q32854 Invitrogen, Waltham, MA, USA) measurement. The libraries were subjected to bead-based normalization and were sequenced using V2 sequencing reagent kits on a NextSeq550 platform (Illumina, San Diego, CA, USA) as per manufacturer recommendations.

### 2.6. NGS Variant Calling and Data Analysis

The raw sequence reads FASTQ files were converted to BAM and VCF files using Qiagen clinical insight-interpret (QCI-I) clinical decision support software (Qiagen, Germantown, MD, USA). The VCF files were analyzed using QCI-I for SNVs and small indels/duplications. Variants with a variant allele frequency (VAF) >5% and a total read depth of >250X were filtered for analysis. The variants were categorized using evidence-based literature and manually-curated data in QCI-I into tier classification based on AMP guidelines for classifying somatic variants. The variants were compared for concordance with previously reported variants, as the same samples were sequenced on a 54 gene myeloid panel and reported through PierianDx reporting solution.

### 2.7. Analytical Comparison between 523-Gene NGS Panel and 54-Gene NGS Panel

Of the 30 samples included in this study, the first 15 samples were analyzed with both the 54-gene standard panel and 523-gene NGS panel, while the later 15 samples were clinically reported with the 523-gene NGS panel. Overall, 22 SNVs were compared for concordance in the first 15 cases, while all 30 cases were included to calculate additional findings. In the 15 cases analyzed only with the 523-gene NGS panel was an additional finding considered if the pathogenic variant was detected in a gene not covered in the 54-gene panel.

### 2.8. OGM and Sequencing Data: Compound Heterozygous Events in NxClinical Software

The current iteration of NxClinical does not support the SV calls from OGM data; hence Bionano access (v.1.6) was utilized for OGM data, QCI-I for sequencing data, and NxClinical for combined CNV and sequencing data viewing and analysis of compound heterozygous events overlapping CNV segments. In addition to simultaneous visualization of CNV and sequencing variants, BAM files from the NGS panel were utilized to call CNVs in NxClinical using the baseline sequencing reads throughout the genome. This approach was used to confirm the novel CNV calls detected by OGM with an alternative method (NGS) and detect compound heterozygous events.

## 3. Results

### 3.1. OGM Quality Control Metrics and Variant Filtering

A typical run for OGM included the processing of eight samples in a batch for DNA isolation, and 12 samples for labeling, while three samples were loaded into one nano-channel chip onto the Saphyr instrument at a given time. All 30 samples passed the quality control metrics with an average N50 (>150 kb) of 300 kb (±37), map rate of 87.6% (±6.8), label density of 15.9/100 kb (±1.2), and average coverage of 391x (±96). In total, 44,096 SVs were identified in 30 samples, with an average of ~1469 SVs per sample. Of all the identified SVs, a total of 1825 SVs remained after filtration (96.6% variants filtered out), with an average of ~60 SVs per sample that were further interrogated. 

### 3.2. OGM Results: Concordance, Higher Resolution/Resolving Identified Events, and Additional Findings

OGM achieved a 100% concordance with karyotyping and FISH in identifying all clinically reported SVs in all 30 cases. The 30 cases included 22 simple cases (<3 aberrations) and eight complex cases (>3 aberrations), based on aberrations detected with karyotyping. OGM achieved 100% concordance in identifying the 72 SVs that included 31 aneuploidies, 16 deletions, 10 translocations, five insertions/duplication/additional material, nine marker chromosomes, and one ring chromosome (Supplementary File S1).

In the 22 simple cases, seven cases were negative with no reported SV with Karyotyping and FISH. OGM detected cytogenetic aberrations in 4/7 (57%) cases, detecting nine SVs that included three translocations, one inversion, and five CNVs. Further 5/22 (22%) cases were re-classified as complex (>3 aberrations) with OGM. 

**Figure 2:**
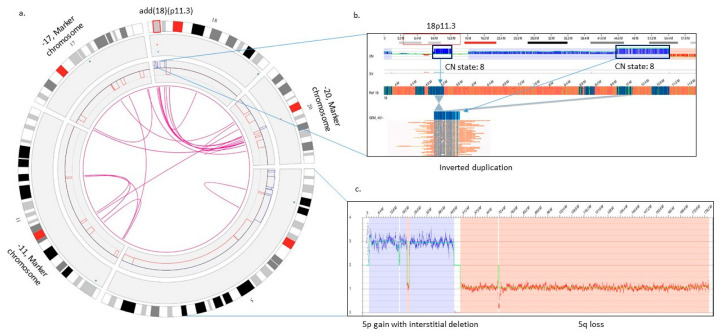
Optical genome mapping resolves a complex case of AML with higher resolution as compared to karyotype {45,XY,-5,-11,-17,add(18)(p11.3),-20,+3mar[19]/46,XY[1]} and FISH. (**a**) The circos plot summarizing the SVs identified in the genome: copy number gain on 5p, copy number loss on 5q, amplification on 18p, and complex rearrangements on chromosomes 11, 17, and 20. Optical genome mapping identifying the identity of marker chromosomes as 11, 17, and 20. (**b**) Zoomed-in view of 18p showing the amplification and complex rearrangement at 18p11.3 with a CN state of 8 of both the fused regions of the genome. (**c**) Zoomed in view of chromosome 5 showing copy number gain on 5p with an interstitial deletion and a copy number loss on 5q.

In the eight complex cases, OGM achieved 100% concordance in identifying all clinically relevant aberrations, while resolving previously observed aberrations with higher resolution. For example: in a case of AML (GEM-451) with a complex karyotype of 45,XY,-5,-11,-17,add(18)(p11.3),-20,+3mar[19]/46,XY[1], with loss of 5q and gain of 5p by FISH, OGM identified gain of 5p, loss of 5q, complex rearrangements on chromosomes 11, 17, and 20 with several intra- and inter-chromosomal translocations (thus, resolving-11, -17, -20, and identifying the three marker chromosomes as 11, 17, and 20), and add(18)(p11.3) as an amplification at 18p11.3 with a CN state of 8 (Figure 2a–c).

**Figure 3:**
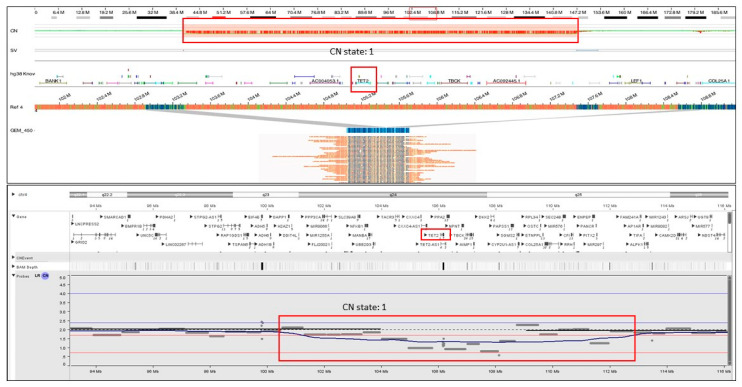
Optical genome mapping identifying a copy number loss on chr 4 [4q24q25(103311803_107478954)x1) deleting the *TET2* gene, which was missed by karyotyping. The CNV signals from the sequencing data visualized in the NxClinical software were indicative of the copy number loss and confirmed the deletion detected by optical genome mapping.

**Figure 4:**
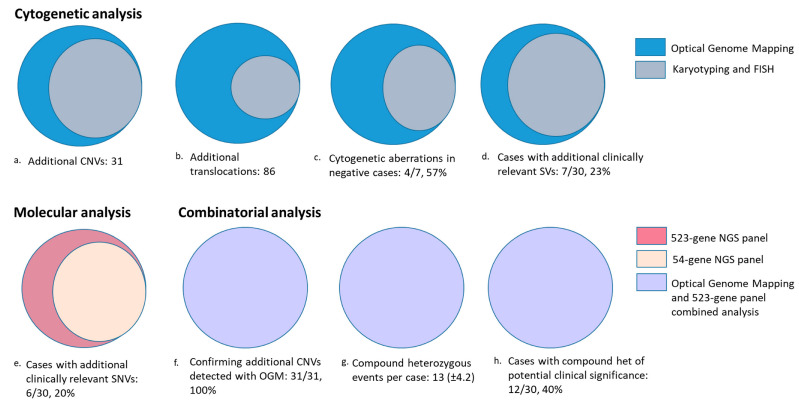
Charts representing additional findings with the proposed workflow (techniques and analysis) compared to standard-of-care methods.

In addition to the previously identified SVs and CNVs, OGM identified 86 additional translocations and 31 copy number changes (Supplementary File S1). Of these 117 additional SVs, 10 SVs were detected in seven cases (7/30, 23%), of which five SVs detected in five cases (16.6%) were aberrations listed in NCCN guidelines, and five SVs in four cases (12.9%) were aberrations listed in NHS guidelines for hematological neoplasms, demonstrating the utility of OGM in identifying clinically relevant information as compared to SOC methods. Although the translocations were not validated (beyond the scope of this study), signals for all 31 additional CNVs could be visualized using the sequencing data in the NxClinical software (Figure 3 and Figure 4a–d).

### 3.3. 523-Gene NGS Panel Quality Control Metrics

A typical sequencing run of the 523 gene NGS panel performed on the NextSeq550 platform consisted of 10 samples, with the 30 samples included in this study run across seven different runs. The average percentage reads passing filter (PCT_PF) for a typical run was observed to be >90%. The percent base calls with a quality score of Q30 or higher for read 1 and 2 were >90%. The four critical DNA library QC parameters viz. the median insert size from the sequencing reads for the runs was found to be approximately 125 bp; the average usable MSI counts were found to be 120; the percent of exon bases with coverage > 50X and percent target bases with coverage > 250X were found to be >99 and >95, respectively, for all the runs. The variants were filtered in QCI-I using a built-in decision tree, filtering for rare somatic variants with a VAF ≥ 5%, read depth (>250x), and classified into tier-based classification, with the analyst and a board-certified director reviewing each call and classification.

### 3.4. 523-Gene NGS Panel: Concordance and Additional Findings

The 523-gene NGS panel achieved 100% concordance in identifying the 22 previously identified clinically relevant sequence variants in these 15 samples. QCI-I was 100% concordant in classifying the variants into correct tier-based classification compared to expertly classified reported variants. Further, the panel was able to identify nine additional clinically relevant variants (three tier 1 variants and six tier 2 variants) in six samples (6/30, 20%) (Supplementary File S2) (Figure 4e).

### 3.5. OGM and 523-Gene NGS Panel: Compound Heterozygous Events

The simultaneous visualization of CNVs and sequencing variants (SNV and small indels) from OGM and 523-gene NGS panel, respectively in the NxClinical software was utilized to detect clinically relevant compound heterozygous events. In the 30 samples, the complementary analysis identified 324 compound heterozygous events, with 10 (±3.2) events per sample. Of the 324 events, 13 events of potential clinical relevance were detected in 12 cases (12/30, 40%), of which, four events detected in four cases (4/30 13.3%) were impacting genes included in the NCCN guidelines, while nine events detected in nine cases (9/30, 30%) were impacting genes implicated in the hematological malignancies (genes not included in NCCN or NHS guidelines) (Figure 4f–h). The details of the events are listed in Supplementary File S3.

**Figure 5:**
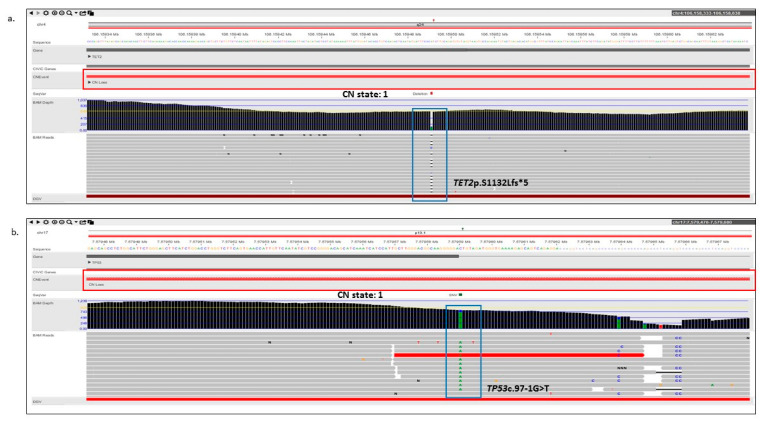
Representative examples of compound heterozygous events in two samples detected with combined visualization of copy number variants and single nucleotide variants in software. (**a**) The copy number loss is highlighted in red, with a CN state of 1, while the pathogenic TET2p.S1132Lfs*5 variant is highlighted in blue. (**b**) The copy number loss is highlighted in red, with a CN state of 1, while the pathogenic *TP53*c.97-1G>T splice variant is highlighted in blue.

For example, in a case of MDS (sample 6), OGM detected a heterozygous interstitial deletion on chr 4 [4q24q25(103311803_107478954)x1], which included the *TET2* gene, while the sequencing data detected a pathogenic frameshift variant (*TET2*p.S1132Lfs*6, Tier 2C classification) on the other allele (Figure 5a). Further, the deletion on chromosome 4 detected by OGM was also confirmed using the sequencing reads (Figure 2a–b). It must be noted that this cryptic deletion cannot be identified by karyotyping and is not part of the targeted FISH panel. In the case of AML (sample 7), OGM identified a complex rearrangement on chromosome 17 involving the deletion of the *TP53* gene, while NGS detected a pathogenic splice site alteration (*TP53*c.97-1G>T; Tier 1A classification) (Figure 5b).

## 4. Risk Stratification

Risk stratification was performed for AML and MDS cases based on the European LeukemiaNet (ELN) risk stratification and the Revised International Prognostic Scoring System (IPSS-R) cytogenetic risk groups guidelines, respectively. For risk stratification, ≥3 aberrations with OGM (≥5 Mb CNV, inversions and translocations) were considered as a complex cytogenetic profile. There was no change in the AML cases; however, the IPSS-R risk grouping changed from very good/good to very poor in 22% (2/9) of cases based on cytogenetic analysis alone (karyotyping and FISH vs. OGM), where OGM identified a complex cytogenetic profile (Samples 430 and 450; Supplementary File S1).

## 5. Discussion

The present study aimed at evaluating a combinatorial approach of utilizing OGM and the 523-gene NGS panel as an alternate diagnostic workflow compared to the current SOC cytogenetic and molecular technologies for genomic characterization of myeloid cancers. A similar approach was recently used by Nilius-Eliliwi et al., 2022, in the characterization of a case of AML using OGM and whole exome sequencing, identifying novel gene fusion and rare variants, proposing a next-generation diagnostic workflow. The present study demonstrated the following: (a) higher resolution of OGM in detecting SVs and CNVs as compared to the combination of current SOC cytogenetic techniques (karyotyping and FISH), (b) greater clinical utility of the 523-gene NGS panel as compared to a 54-gene NGS panel for detecting clinically relevant sequencing variants, and (c) greater simplicity and clinical utility of combining OGM with a 523-gene NGS panel for a comprehensive approach to a higher success rate in the profiling of myeloid cancers.

### 5.1. Cytogenomics: OGM Compared to Standard-of-Care Technologies (Karyotype and FISH)

The sample processing for OGM is simple and standardized for BMA and blood samples and can be employed by diagnostic molecular or cytogenetic laboratories for clinical implementation. Typically, 16 samples were isolated in a single day in two batches of eight samples, each, and 12 samples were labelled in a single batch. Three samples were loaded on a single Saphyr chip and imaged on the instrument in a single run, with ~1500 gigabases of data collected for each sample enabling an average depth of coverage of ~391x. All 30 samples passed the pre-analytical and analytical QC metrics, which highlights the ease of use and demonstrates the feasibility of processing blood and BMA samples for cytogenetic analysis in a routine laboratory. The entire OGM workflow from sample to SV calls can be accomplished in ~4–5 days.

OGM achieved 100% concordance in detecting clinically relevant SVs and CNVs in the cases with simple cytogenetic profiles, while demonstrating a higher resolution in resolving the genetic aberration in cases with a complex cytogenetic profile. These results agree with recently published work on the characterization of hematological malignancies using OGM [15,16,17,18,20]. The cytogenetic profile obtained with karyotyping and FISH in complex cases could not identify or detect the cryptic, clinically relevant aberrations, while it was only after the analysis with OGM that the “true” complex nature of these genetic aberrations was revealed. As recently demonstrated, we found that OGM could discern complex rearrangements and resolve previously identified genetic aberrations with much higher resolution. OGM could resolve the identity of all 10 marker chromosomes in complex cases.

Importantly, OGM detected 117 additional cytogenetic aberrations that included 31 CNVs and 86 translocations. Notably, the cytogenetic classification changed from simple to complex in 22% (5/22) of cases, while OGM detected cytogenetic aberrations in 57% (4/7) of cases that were previously cytogenetically normal. Overall, the IPSS-R cytogenetic risk group changed from very good/good to very poor in 22% of MDS cases, with additional clinically relevant SVs in 23% (7/30) cases, demonstrating its incremental clinical utility compared to current SOC methods.

Overall, OGM offers several advantages over karyotyping and FISH for genomic characterization of myeloid cancers. OGM demonstrated the unique ability for genome-wide analysis of all classes of SVs and CNVs at high resolution in a single assay, which enabled the detection of additional potential clinically relevant SVs.

### 5.2. Molecular Profiling: 523-Gene NGS Panel Compared to 54-Gene NGS Panel

The comprehensive molecular profiling of myeloid cancers is of significant interest and owing to the decreasing cost of sequencing technologies, it is now possible to evaluate and implement comprehensive gene panels, or genome sequencing (GS) for clinical care. However, because of data analysis and reporting complexities, limited gene (≤54 genes) panels have been routinely employed by clinical laboratories. As we previously demonstrated [7], the 523-gene NGS panel achieved excellent quality control metrics and demonstrated a 100% concordance in identifying clinically relevant variants, previously identified with the 54-gene panel. In addition, we demonstrate the ability of a larger gene panel in detecting additional clinically relevant sequencing variants as evident from novel findings in 20% (6/30) of cases compared to the 54-gene NGS panel.

Overall, the 523-gene panel has several advantages as compared to the 54-gene panel which include the following: (a) the ability to detect additional clinically relevant variants in genes currently not covered in smaller gene panels, and (b) the 523-gene panel is a comprehensive pan-cancer panel that includes genes implicated across cancers and has the potential to identify variants that would potentially affect the diagnostic, prognostic, and/or therapeutic course for management of these malignancies. A possible caveat with comprehensive gene panels is the discovery of variants in genes of unknown significance. However, when pooled together in common databases, this could lead to recognition of unique variants that may have unique biological, prognostic, and/or therapeutic significance. Molecular genetic or pathology laboratories are already equipped with tools for sequencing small gene panels and are familiar with library preparation and sequencing, hence the adoption of a larger gene panel would not lead to any major changes to the lab workflow.

### 5.3. OGM and 523-Gene NGS Panel Compared to the Current Diagnostic Workflow (Karyotype, FISH, and 54-Gene NGS Panel)

**Figure 6:**
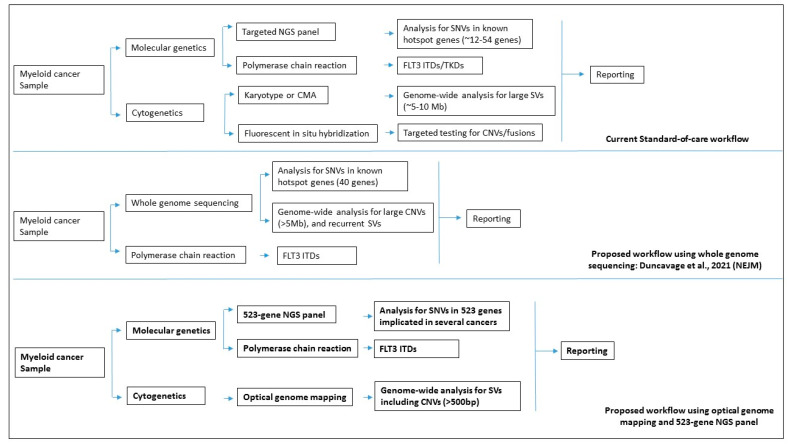
Comparison of the proposed workflow of optical genome mapping and 523-gene NGS panel with current diagnostic workflow and workflow proposed by Duncavage et al., 2021 [19] using whole genome sequencing.

The combination of OGM and the 523-gene NGS panel demonstrates several advantages as compared to current SOC technologies. Apart from the higher resolution and comprehensive genome-wide coverage of SVs via OGM [16,21], the simultaneous use of these technologies enables rapid detection of compound heterozygous events that had not been interrogated with routine methods [22]. The combinatorial analysis identified compound heterozygous events of potential clinical significance in 20% (6/30) of cases. These events impacted the *TET2* and *TP53* gene, both of which are included in NCCN guidelines. *TET2* is an important gene implicated in MDS and is routinely included in the NGS panels for detecting sequencing variants. In one case, OGM uniquely identified a large deletion of 4.1 Mb (missed by karyotyping and not interrogated by FISH), and NGS identified an LoF variant (detected by both small and large panels). By visualization in the NxClinical platform, observation of both variants allowed easy determination of compound heterozygosity involving this gene in the tumor (Figure 5a). In an AML case, however, loss of chromosome 17 (indicative of loss of *TP53*) was detected by karyotyping, which was resolved as a complex rearrangement of chr 17 with the loss of one copy of *TP53* gene by OGM. The 523-gene NGS panel identified a splice variant in this gene that was missed by the 54-gene NGS panel. By visualization in the NxClinical platform, observation of both variants allowed easy determination of compound heterozygosity involving this gene in the tumor (Figure 5b). Resolving the genomic complexity in these cases highlights the benefit of obtaining clinically relevant information, on top of their respective advantages. Further, the additional CNVs detected by OGM were confirmed with the copy number signals in NxClinical using the sequencing data. Notably, the 523-gene NGS panel does not have a genome-wide coverage and thus, the sequencing data could only be indicative of copy number changes that can be used to confirm the calls originally detected by OGM. Overall, these two technologies complement each other and enable comprehensive and robust profiling of myeloid cancers in clinical care (Figure 6).

### 5.4. OGM and 523-Gene NGS Panel Compared to GS for Myeloid Cancers

A recent study demonstrated the use of GS to detect both cytogenetic (karyotype level) and sequencing variants in a single assay in cases with AML, as compared to karyotyping and a 40-gene NGS panel [19]. The results of GS were encouraging but several challenges need careful consideration before implementation for clinical use. On comparing GS with MyeloSeq (40 gene NGS panel), GS at ~50x depth failed to detect several actionable sequence variants across a wide range of VAF (false-negative results). Upon increasing GS coverage depth (~120x), better performance was achieved but an important *KRAS* variant (detected with Myeloseq at 5.2% VAF) was still missed. The failure was attributed to the lower depth of coverage of GS (~120x vs. 500x), compared with MyeloSeq. We consider that the 523-gene panel provides an ideal alternative that achieves coverage of >250x across the targeted genes and provides a comprehensive assessment of sequencing variants without compromising clinically relevant variants present at low VAF, representing the heterogeneous tumor burden in these malignancies. For cytogenetic characterization, GS at ~50x depth was concordant in detecting CNVs and translocations detected with karyotype at the 5 Mb resolution (LoD for karyotype). However, the data analyzed by GS was limited to interrogating recurrent SVs only and did not address important multi-partner translocations such as those involving *IGH* and *KMT2A*. Non-recurrent SVs, CNVs smaller than 5 Mb, and cases with complex rearrangements/marker chromosomes were not investigated or reported by GS. Given that ~50% of myeloid cancers are considered cytogenetically normal when assessed by standard karyotyping, GS (at the current depth of coverage and exhaustive bioinformatics capabilities needed for SV detection) does not offer additional benefits over the traditional cytogenetic methods. Multiple studies have demonstrated that OGM can detect clinically actionable SVs and novel fusions that can affect the risk stratification and management of these cancers [15,16,17,18,21,22]. Overall, the combined cost for OGM and the 523-gene NGS panel is lower than GS at 50x coverage and offers several advantages with regard to detecting clinically relevant variants, data analysis, and reporting [16] (Table 1, Figure 6). Based on current data and observations from other studies, GS at 50x or 120x depth will continue to miss sequence variants that are readily detected by high-depth gene panels as well as structural variations that are intractable to GS, while the OGM + 523 gene panel will detect all variants targeted by SOC panels as well as additional clinically relevant variants.

## 6. Limitations of the Study

The study was performed on a limited number of cases and a validation of this workflow with a higher number of samples is needed. The additional translocation (novel fusion event) identified in this study requires validation and the functional implications of novel gene fusions need to be investigated. Even though this was beyond the scope of our study, we have shared all relevant information for future discovery. Despite these limitations, this proof-of-principle study demonstrating the use of OGM and 523-gene NGS panel for comprehensive genomic profiling of myeloid neoplasm shows immense potential to be integrated into a routine cytogenetic laboratory for maximum impact on disease diagnosis and management.

## 7. Conclusions

This study demonstrates the higher sensitivity, resolution, accuracy, and ability to reveal cryptic and clinically relevant novel variants in myeloid cancers as compared to SOC methodologies. This cost-effective approach of using OGM and a 523-gene NGS panel for comprehensive genomic profiling of myeloid cancers has the potential to not only increase the yield of actionable targets leading to improved clinical outcomes but also to help resolve the ongoing conundrum of apparently genomically normal myeloid cancers by identifying cryptic aberrations.

## Figures and Tables

**Table 1:** Comparison of the proposed workflow of optical genome mapping and 523-gene NGS panel with current diagnostic workflow and workflow proposed by Duncavage et al., 2021 [19] using whole-genome sequencing. _: not applicable, √: detected, √* small variants, √# large variants, ^ denovo pipeline.

Variant Classification	Variant Types	Variant Classes	Current Diagnostic Workflow			Duncavage et al., 2021 [19]	Proposed Workflow	
			Karyotype	FISH	54-Gene NGS Panel	Short-Read WGS	OGM	523-Gene NGS Panel
Sequencing variants and coverage	SNVs	_	_	_	>500x	50x (probability of false negatives for variants detected at low VAF with 500x panels)	_	>250x
	Indels	_	_	_	√*	√*	√#	√*
Aneuploidy	Monosomy	Monosomy	√	Targeted FISH probes	_	√	√	_
	Trisomy	Trisomy	√	Targeted FISH probes	_	√	√	_
	Triploidy	Triploidy	√	Targeted FISH probes	_	√	no	_
	Tetraploidy	Tetraploidy	√	Targeted FISH probes	_	√	no	_
	Ring chromosome	Ring chromosome	√	No	_	No	≥500 kbp + fusion break	_
Copy Number Variants	Deletions/Duplication	Interstitial	5 Mb or larger	Targeted FISH probes	_	5 Mb or larger	≥5 kbp	_
		Terminal	5 Mb or larger	Targeted FISH probes	_	5 Mb or larger	≥5 kbp	_
	Insertion	Interstitial (unknown sequence)	5 Mb or larger	No	_	No	~5 kbp	_
					_			_
Structural variants	Translocations	Balanced translocations	√	Dependent on FISH probes	_	Only recurrent translocations were investigated with current bioinformatics processing	√	_
		Unbalanced translocations	√	Dependent on FISH probes	_	Only recurrent translocations were investigated with current bioinformatics processing	√	_
	Inversions	Pericentric			_	No	√	_
		Paracentric	5 Mb or larger	No	_	No	≥30 kbp	_
Homozygosity mapping	LOH	AOH/ROH/LOH	No	No	_	√	25 mb ^	_
Microsatellite/Macrostaellite repeats	Repeats	Expansions/Contractions	No	No	_	Limited to short repeats	≥500 bp	_

## Data Availability

The data presented in this study are available in this article and supplementary material.

## Supplementary Materials

The following supporting information can be downloaded at: https://www.mdpi.com/article/10.3390/cancers15123214/s1, File S1: Comparison of Optical Genome Mapping and Standard of care cytogenetic methods (Karyotyping and FISH); File S2: Comparison of 523-gene panel and 54-gene panel; File S3: Compound heterozygous events.
